# Dopamine modulates the retinal clock through melanopsin-dependent regulation of cholinergic waves during development

**DOI:** 10.1186/s12915-023-01647-6

**Published:** 2023-06-26

**Authors:** Chaimaa Kinane, Hugo Calligaro, Antonin Jandot, Christine Coutanson, Nasser Haddjeri, Mohamed Bennis, Ouria Dkhissi-Benyahya

**Affiliations:** 1 Inserm, Stem Cell and Brain Research Institute U1208, Univ Lyon, Université Claude Bernard Lyon 1, 18 Avenue du Doyen Lépine, 69500 Bron, France; 2 Laboratory of Pharmacology, Neurobiology, Anthropobiology and Environment, University Cadi Ayyad, Marrakech, Morocco; 3 Salk Institute for Biological Studies, La Lolla, CA USA

**Keywords:** Circadian rhythms, Dopamine, Melanopsin, Acetylcholine, Retinal waves, Light

## Abstract

**Background:**

The mammalian retina contains an autonomous circadian clock that controls various aspects of retinal physiology and function, including dopamine (DA) release by amacrine cells. This neurotransmitter plays a critical role in retina development, visual signalling, and phase resetting of the retinal clock in adulthood. Interestingly, bidirectional regulation between dopaminergic cells and melanopsin-expressing retinal ganglion cells has been demonstrated in the adult and during development. Additionally, the adult melanopsin knockout mouse (*Opn4* ^*−/−*^) exhibits a shortening of the endogenous period of the retinal clock. However, whether DA and / or melanopsin influence the retinal clock mechanism during its maturation is still unknown.

**Results:**

Using wild-type *Per2* ^*Luc*^ and melanopsin knockout (*Opn4* ^*−/−*^::*Per2* ^*Luc*^) mice at different postnatal stages, we found that the retina generates self-sustained circadian rhythms from postnatal day 5 in both genotypes and that the ability to express these rhythms emerges in the absence of external time cues. Intriguingly, only in wild-type explants, DA supplementation lengthened the endogenous period of the clock during the first week of postnatal development through both D1- and D2-like dopaminergic receptors. Furthermore, the blockade of spontaneous cholinergic retinal waves, which drive DA release in the early developmental stages, shortened the period and reduced the light-induced phase shift of the retinal clock only in wild-type retinas.

**Conclusions:**

These data suggest that DA modulates the molecular core of the clock through melanopsin-dependent regulation of acetylcholine retinal waves, thus offering an unprecedented role of DA and melanopsin in the endogenous functioning and the light response of the retinal clock during development.

**Supplementary Information:**

The online version contains supplementary material available at 10.1186/s12915-023-01647-6.

## Background

Dopamine (DA) is a critical neurotransmitter in the retina, synthesized and released by a small population of retinal amacrine cells. DA plays a crucial modulatory role in both daily and circadian retinal functions, including phase resetting of the retinal circadian oscillator [1], light adaptation and contrast sensitivity [2, 3], regulation of clock genes [4], gap junction coupling between photoreceptor, amacrine and ganglion cell [5–7] and eye development [8, 9]. DA triggers this wide array of intracellular changes via two metabotropic DA receptor (DR) families, the D1 (including D1R and D5R) and the D2 types (D2R, D3R, and D4R), which are both G protein-coupled receptors. Activation of D1 receptors stimulates cAMP production and protein kinase A activity. In contrast, activation of D2 type negatively regulates cAMP, decreasing protein kinase A activity [10]. Most retinal cells, including Müller glial cells, can be modulated by DA as they express at least one type of dopaminergic receptor. In rodents, rods and cones express D4Rs and may express D2Rs in some species [11, 12], whereas horizontal, bipolar, amacrine, and ganglion cells mainly express D1Rs [13–18]. DA is assumed to act on the retinal circadian clock through D1Rs, whereas D2Rs play a role in the induction of *Per* clock genes by light [4, 6, 19, 20].

In the adult, the mammalian retinal clock consists of an integrated network of clocks localized throughout retinal cells [21–25] which give rise to the rhythmic regulation of the physiology and function of this tissue. However, although clock gene expression has recently been reported at embryonic stages in the mouse eye [26], it is still unknown when retinal cells first express circadian rhythms and how they synchronize during development. Interestingly, we have previously shown that the adult melanopsin knockout mouse (*Opn4* ^*−/−*^) exhibits a shortening of the endogenous period of the retinal clock and dysfunction of the retinal dopaminergic system [21, 27], suggesting that DA and /or melanopsin represent potential candidates for influencing the maturation and the endogenous functioning of this clock. Indeed, DA synthesis and release are regulated by the retinal circadian clock [28–30] and dopaminergic cell activity is modulated by melanopsin intrinsically photosensitive ganglion cells (ipRGCs) [31–33]. These ipRGCs stratify with dopaminergic cells during both development and adulthood, conveying retrograde signaling to the inner retina [34–36]. In the current study, we established for the first time the onset and fundamental features of the retinal clock during development in the wild-type *Per2* ^*Luc*^ mouse. We then investigated whether the absence of melanopsin affected the ontogeny and the maturation of the retinal clock using the *Opn4* ^*−/−*^::*Per2* ^*Luc*^ mouse and analyzed the potential role of DA in these processes. Interestingly, several pieces of evidence suggest that ipRGCs and cholinergic retinal waves, which consist of periodic spontaneous bursts of ganglion cell activity [37, 38] observed from postnatal day 1 (P1) to P10-P11 [39, 40] interplay through DA. In particular, ipRGCs modulate the circuitry of cholinergic waves in the neonatal retina, even in darkness [41]. On the other hand, in the absence of these waves, dopaminergic signaling regulates the extent of ipRGCs gap junction coupling during development [7, 42]. Therefore, we sought to identify whether cholinergic waves have an impact on the endogenous and light-induced response properties of the retinal clock.

## Results

### Circadian oscillations of PER2::Luc is first detected in vitro at postnatal day 5

**Fig. 1:**
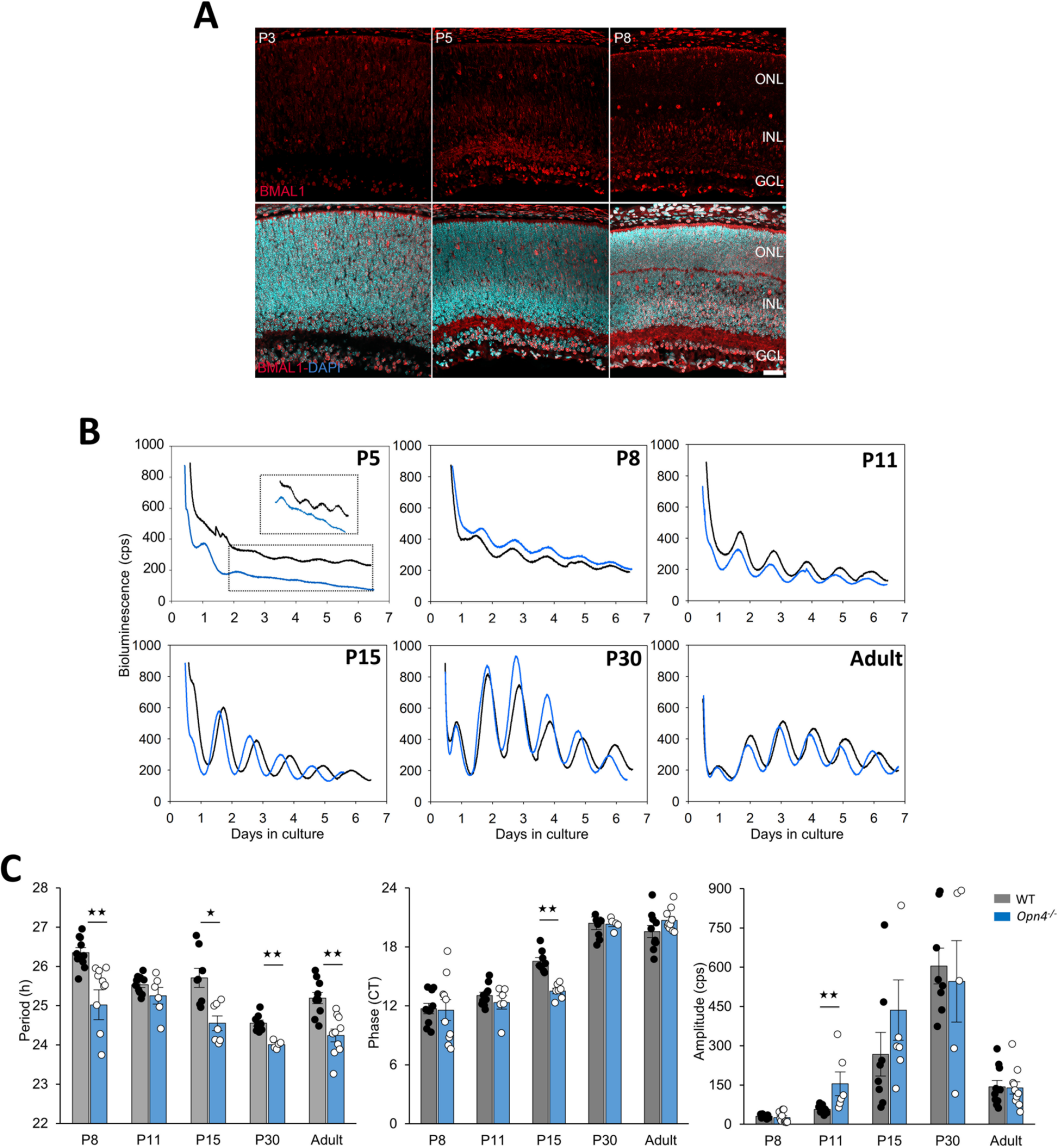
Expression of BMAL1 and ontogenesis of PER2::Luc circadian oscillations in wild-type and *Opn4* ^*−/−*^ retinal explants. **A** Immunostaining of BMAL1 (red) in retinal sections of wild-type mice counterstained with DAPI (blue) at P3, P5 and P8. Scale bar = 10 µm: NBL, neuroblastic retina; ONL, outer nuclear layer; INL, inner nuclear layer and GCL, ganglion cell layer. **B** Representative bioluminescence recording of PER2::Luc retinal explants from *Per2* ^*Luc*^ (black line) and *Opn4* ^*−/−*^ *::Per2* ^*Luc*^ (blue line) mice at different postnatal stages (P5, P8, P11, P15, P30) and in the adult. Retinal explants were cultured for at least 6 days without medium change. The dotted black rectangle corresponds to an enlargement of bioluminescence traces of both genotypes at P5. **C** Means of the endogenous period, the circadian phase, and the amplitude in both genotypes during development. The total numbers of retinas used were respectively for wild-type mice: P8, *n* = 10; P11, *n* = 10; P15, *n* = 6; P30, *n* = 8; adult, *n* = 10 and for *Opn4* ^*−/−*^ *::Per2* ^*Luc*^; P8, *n* = 6; P11, *n* = 6; P15, *n* = 7; P30, *n* = 6 and adult, *n* = 10. Bars represent the mean ± SEM (ANOVA, ** = *p* ≤ 0.01; *** = *p* ≤ 0.001)

Retinal explants from different developmental stages, including embryonic day 18 (E18, Additional file 1: Fig. S1) and postnatal days (P) 1, 5, 8, 15 and 30 as well as adult (P60-90) *Per2* ^*Luc*^ mice, were continuously recorded for at least six days without medium change and under constant dark conditions (Fig. 1). A rhythmic expression of PER2::Luc was first observed in P5 retinal explants after 2 days in vitro, which became more robust with higher amplitude, defined as the difference between the peak and the mesor (for midline estimating statistic of rhythm) from P8 to P30 (Fig. 1B). To determine from which cells this signal arises, wild-type retinal cryosections were labelled with an antibody directed against the core clock protein BMAL1 at early postnatal stages P3, P5 and P8 (Fig. 1A). A few BMAL1-positive cells were observed in the outer part of the neuroblastic retina and in the ganglion cell layer (GCL) at P3. At P5 and P8, the number of BMAL1-positive cells increased mainly in the inner part of the inner nuclear layer (INL) and in the GCL. During retinal maturation, the endogenous period (the time from peak to peak of PER2::Luc oscillations) was significantly shortened between P8 and P11 (from 26.35 ± 0.12 h to 25.54 ± 0.07 h; *p* = 0.00038), and between P15 and P30 (from 25.71 ± 0.24 h to 24.55 ± 0.08 h; *p* = 0.0011; Fig. 1C) in wild-type retinal explants. Then, the period was lengthened in the adult (25.19 ± 0.14 h; p = 0.0058). The phase (the circadian time of the first peak of PER2::Luc expression) remained constant from P8 to P11 (*p* = 0.069), is delayed from P11 to P15 (respectively CT 13.05 ± 0.35 and CT 16.54 ± 0.39; *p* = 0.0003), from P15 to P30 (CT 20.40 ± 0.65; *p* = 0.0003) and remained unchanged at a later stage (*p* ≥ 0.05). Finally, the amplitude of PER2::Luc oscillations increased from P8 to P30 (P8: 30.51 ± 2.04 counts per second (cps); P11: 56.34 ± 4.58 cps; P15: 267.60 ± 83.42 cps and P30: 604.80 ± 68.50 cps; *p* ≥ 0.01) followed by a significant reduction in the adult (143.43 ± 23.85 cps; *p* = 0.00004).

### The absence of melanopsin shortens the period of the retinal clock

As we previously reported a shortening of the endogenous period of the retinal clock and a loss of clock gene rhythms in the photoreceptor layer in the adult *Opn4* ^*−/−*^ mouse [21, 27, 43], we analyzed the potential impact of the absence of melanopsin on the onset and the maturation of the retinal clock. Similar to the wild-type mouse, PER2::Luc oscillations started at P5 in the *Opn4* ^*−/−*^::*Per2* ^*Luc*^ mouse (Fig. 1B). However, the amplitude was lower compared to the wild-type mouse, and did not allow reliable analysis of the clock properties at this developmental stage. PER2::Luc oscillations then became more robust, with mostly similar amplitudes between genotypes in the different developmental stages. The main difference concerned the significant shortening of the period observed in all developmental stages in *Opn4* ^*−/−*^::*Per2* ^*Luc*^ retinal explants (P8: 25.32 ± 0,24 h, *p* = 0.0011; P15: 24.55 ± 0.19 h, *p* = 0.01; P30: 24.01 ± 0.04 h, *p* = 0.0034; adult: 24.24 ± 0.16 h; *p* = 0.0011, Fig. 1C) suggesting that the absence of melanopsin has a permanent consequence on the period length of the retinal clock. A significant advance in the phase of PER2::Luc oscillations (CT 13.51 ± 0.28; *p* = 0.0011) was observed only at P15 in retinal explants from *Opn4* ^*−/−*^::*Per2* ^*Luc*^ mice compared to wild-type explants (CT 16.54 ± 0.39; *p* = 0.0011). To analyse the relationship between the clock features (period, phase and amplitude) during development, we fitted a linear model function to wildtype and *Opn4* ^*−/−*^::*Per2* ^*Luc*^ data from P8 to P60 (Additional file 2: Fig. S2). For both genotypes, we noticed a negative correlation between the phase and the period during development. This means that as the period become shorter over developmental time, the phase gets delayed (*p* ≤ 0.0001). There was no significant change in the slope between genotypes and across different developmental stages (*p* = 0.25). Additionally, we did not observe a significant correlation between the amplitude of the rhythm and the period for both genotypes (*p* = 0.16).

### Spontaneous PER2::Luc oscillations develop in vitro

**Fig. 2:**
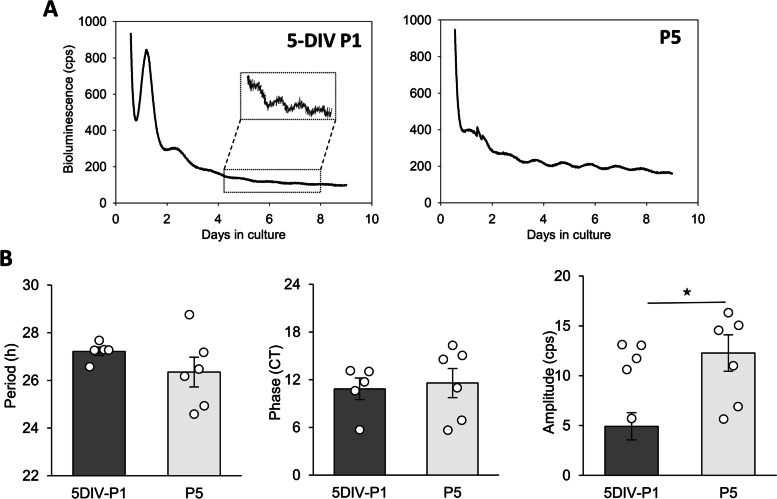
P1 mouse retinal explants develop in vitro. **A** Representative bioluminescence recording of PER2::Luc from P1 and P5 retinal explants. After 5 days in vitro, P1 retinal explants (5-DIV P1) exhibit spontaneous PER2::Luc oscillations. **B** Means of the endogenous period, the phase, and the amplitude of 5-DIV P1 and P5 PER2::Luc retinal explants. The total numbers of retinas used were: 5-DIV P1, *n* = 5, and P5, *n* = 6. Bars represent the mean ± SEM (ANOVA, ** = *p* ≤ 0.01)

**Fig. 3:**
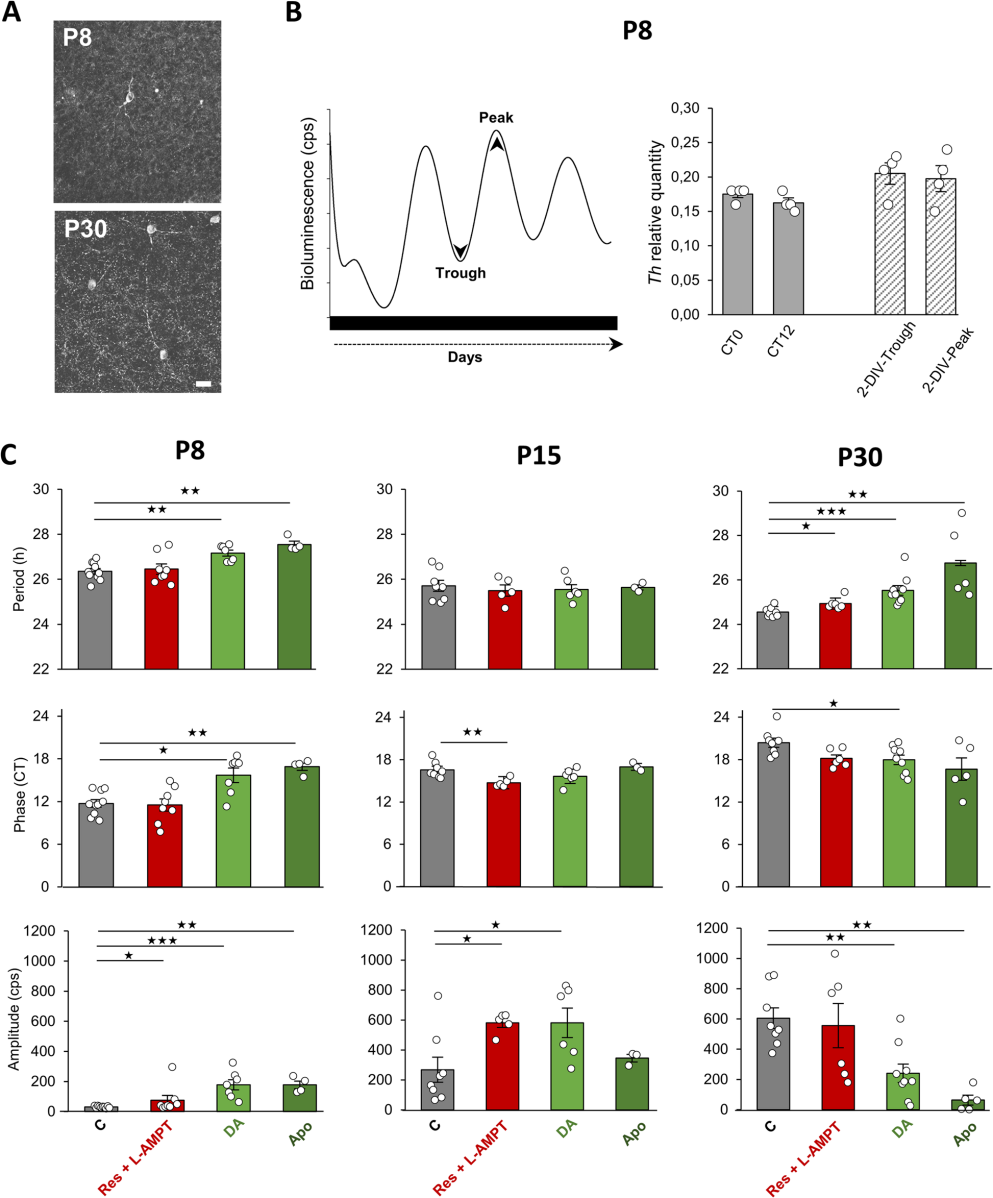
The dopaminergic system in the mouse retina during development. **A** Flatmount retinas showing tyrosine hydroxylase (TH) immunopositive cells in wild-type retinal explants that were cultured for 2-DIV at P8 and P30. Scale bar = 20 µm. **B** Schematic representation of retina sampling at the trough and the peak of PER2::Luc oscillations after 2-DIV (left). Ex vivo (CT0 and CT12) and in vitro relative expression of tyrosine hydroxylase (*Th*) mRNA at P8 (right). **C** Means of the endogenous period, the phase and the amplitude of the retinal clock at 3 postnatal developmental stages (P8, P15 and P30). Retinal explants were supplemented with DA (50 µM), Apo (50 µM), or a combination of Res + L-AMPT (respectively 10 µM and 100 µM) and are compared to non-supplemented control retinas. The total numbers of retinas used were: P8: C, *n* = 10; DA, *n* = 7; Apo, *n* = 4; Res + L-AMPT, *n* = 8); P15: C, *n* = 8. DA, *n* = 6; Apo, *n* = 3; Res + L-AMPT, *n* = 5; P30: C, *n* = 8; DA, *n* = 9; Apo, *n* = 5; Res + L-AMPT, *n* = 6. Bars represent the mean ± SEM (ANOVA, * = *p* ≤ 0.05; ** = *p* ≤ 0.01)

To investigate the development of circadian oscillations in vitro, we cultured retinal explants from P1 *Per2* ^*Luc*^ mice without medium change and under constant dark conditions for 9 days, as oscillations were not detectable before P5 (Fig. 2A, Additional file 3: Fig. S3A). The explants did not exhibit rhythms for the first 4 days in culture, then spontaneous oscillations with low amplitude were observed around 5 days in vitro (5-DIV P1) in all explants. We compared the circadian parameters of 5-DIV P1 explants with P5 cultured retinas (Fig. 3B) and found no differences in the endogenous period (5-DIV P1: 27.22 ± 0.18 h; P5: 26.35 ± 0.62 h; *p* ≥ 0.05), or the phase (5-DIV P1: CT 10.85 ± 1.37; P5: CT 11.58 ± 1.83; *p* ≥ 0.05) between both groups. However, the amplitude was higher in P5 cultured explants (5-DIV P1: 4.92 ± 1.78 cps; P5: 12.27 ± 1.14 cps; *p* = 0.035). Retinal explants from E18 *Per2* ^*Luc*^ embryos were maintained in vitro for more than 6 days but we did not detect any PER2::Luc rhythms (Additional file 2: Fig. S2).

### DA regulates the retinal clock during development in the wild-type mouse

Although the role of DA in the regulation of daily and circadian retinal functions in adult is well known, its role during the ontogeny of the retinal clock has not been investigated. We first examined whether the dopaminergic system analyzed in vitro faithfully reflects in vivo conditions. Immunostaining with an antibody directed against tyrosine hydroxylase (TH), the rate-limiting enzyme of DA synthesis, confirmed that 2-DIV explants retained dopaminergic neurons at P8 and P30 (Fig. 3A). In addition, the relative expression of *Th* mRNA quantified at P8 showed unaltered levels (Fig. 3B: *p* ≥ 0.05) in both ex vivo (CT0 and CT12) and in vitro retinas after 2 days of culture (trough and peak of PER2::Luc oscillations). We then examined whether DA is required for the generation of PER2::Luc rhythms. Retinal explants were treated with both alpha methyl-L-tyrosine (L-AMPT) and reserpine (Res), inhibitors respectively of DA synthesis and vesicular DA uptake (Fig. 3C, red bars) and compared to untreated retinas at P8, P15, and P30 (Fig. 3C, grey bars). At the 3 developmental stages, PER2::Luc rhythms persisted after DA depletion, with no changes in the endogenous period at all stages (P8: 26.45 ± 0.23 h; P15: 25.50 ± 0.25 h; P30: 24.94 ± 0.11 h; *p* ≥ 0.05) by comparison to control retinas of the same stage. Additionally, DA depletion did not affect the phase of PER2::Luc oscillations at P8 (controls: CT 11.73 ± 0.53; depleted-retinas: CT 11.51 ± 0.87; *p* ≥ 0.05) and P30 (controls: CT 20.40 ± 0.65; depleted-retinas: CT18.17 ± 0.50; *p* = 0.053) and induced an advance at P15 (controls: CT 16.54 ± 0.39; depleted retinas: CT 14.73 ± 0.27; *p* = 0.01). The amplitude of the rhythm was slightly increased at both P8 (controls: 30.51 ± 2.04 cps; depleted-retinas 74.72 ± 32.05 cps; *p* = 0.045) and P15 (controls: 267.61 ± 83.42 cps; depleted-retinas: 580.14 ± 30.50 cps; *p* = 0.048). These results suggest that DA is not required for the generation of PER2::Luc rhythm during the retinal clock development. We then investigated whether DA supplementation affects the onset of spontaneous oscillations of PER2::Luc in vitro. In our culture system, the drugs were applied as a bolus and were active for at least 4 circadian cycles, as evidenced by the altered period of PER2::Luc rhythms of supplemented explants compared to untreated retinas. Additional file 3: Fig. S3 shows that P1 explants did not express circadian oscillations in the presence of DA for the first 4–5 days in culture (5-DIV P1 + DA). Furthermore, as observed in the non-supplemented 5-DIV P1 retinas, the oscillations began to emerge after 5 days in vitro with very low amplitudes. No significant difference was observed in the period and the phase between 5-DIV P1 explants supplemented with DA and P5 explants. We then treated explants from the same developmental stages (P8, P15, and P30) with DA (50 µM; light green bars), or apomorphine (Apo), a non-selective DA agonist (Fig. 3C; dark green bars). Interestingly, supplementation with DA or Apo at P8 significantly lengthened the endogenous period (DA: 27.16 ± 0.13 h, *p* = 0.002; Apo: 27.55 ± 0.15 h, *p* = 0.005) and delayed the phase (DA: CT 15.69 ± 1.01, *p* = 0.016; Apo: CT 16.89 ± 0.50, *p* = 0.005) of PER2::Luc oscillations by comparison to non-supplemented explants. Both drugs also lengthened the period at P30 (DA: 25.53 ± 0.22 h, p = 0.0008; Apo: 26.76 ± 0.73 h, *p* = 0.004). The effect of DA supplementation on the amplitude of the PER2::Luc retinal rhythm was variable, with an increase at P8 and P15 (respectively *p* = 0.0007 and *p* = 0.033) and a decrease at P30 (*p* = 0.006). We did not observe any effect of DA supplementation in the period or the phase of PER2::Luc oscillation in adults (respectively: 25.40 ± 0.65 h and CT 18,69 ± 1,21; *p* ≤ 0.05; data not shown).

### The effect of DA on the retinal clock is mediated by both D1 and D2 dopaminergic receptors and gap junctions

**Fig. 4:**
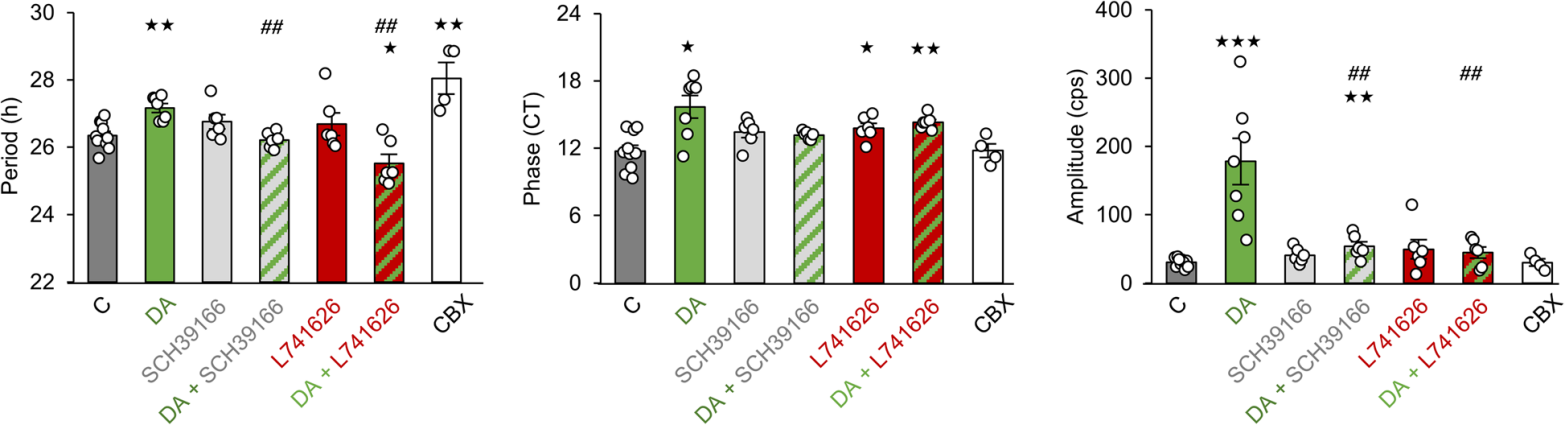
The effect of dopamine on the retinal clock involves both D1- and D2-like receptors. Means of the endogenous period, the phase and the amplitude of P8 retinal explants, supplemented with DA (*n* = 7, 50 µM), a D1R DA antagonist (SCH39166, *n* = 6), a D2R DA antagonist (L741626, *n* = 6), DA combined to SCH39166 (*n* = 6) or L741626 (*n* = 6), or a general gap junction blocker (CBX, *n* = 4) and compared to non-supplemented control retinas (C, *n* = 10). Bars represent mean ± SEM (* = *p* ≤ 0.05; ** = *p* ≤ 0.01; *** = *p* ≤ 0.001, comparison with C group; ^##^ = *p* ≤ 0,01; ^###^ = *p* ≤ 0,001, comparison with DA group)

During development, the period of PER2::Luc oscillations progressively shortened from P8 onwards, and DA supplementation lengthened this fundamental clock parameter at this stage in wild-type mice. To determine which dopaminergic receptors mediate this effect, we focused on this developmental stage. Supplementation of wild-type retinas with the D1R (SCH39166, 50 µM) and the D2R antagonists (L741626; 25 µM) did not alter the period or the amplitude of the oscillations when used alone, except for a slight phase delay observed with L741626 (CT 13.77 ± 0.43, *p* ≤ 0.044; Fig. 4) by comparison with untreated explants. We further combined DA (50 µM) with SCH39166 or L741626 and found that both combinations abolished or significantly reduced the effect of DA on the period of PER2::Luc oscillations (respectively *p* = 0.41 and *p* = 0.026). Furthermore, a period lengthening was observed when P8 retinas were treated with carbenoxolone (CBX, 100 µM), a general gap junction blocker (28.05 ± 0.47 h; *p* = 0.005, Fig. 4).

### DA does not lengthen the period of PER2::Luc retinal rhythms in the *Opn*_*4*_^*−/−*^ mouse during early postnatal development

**Fig. 5:**
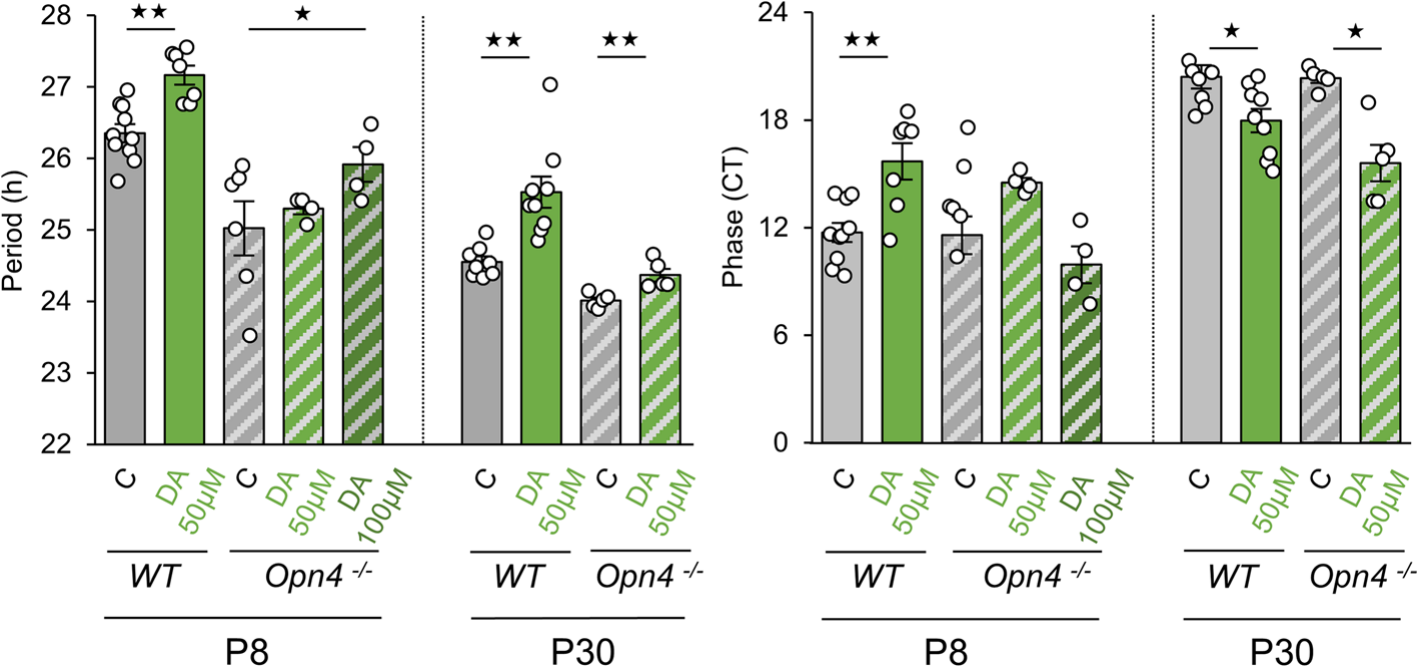
Effect of dopamine on the retinal clock in the absence of melanopsin. Means of the endogenous period and the phase of P8 and P30 retinal explants from *Opn4* ^*−/−*^ *::Per2* ^*Luc*^ mice, supplemented with DA (50 and 100 µM) and compared to non-supplemented control retinas. The total numbers of *Opn4* ^*−/−*^ *::Per2* ^*Luc*^ retinas used were: P8: C, *n* = 6; DA 50 µM, *n* = 4; DA 100 µM, *n* = 4. P30: C, n = 5; DA 50 µM, n = 5. Data from wild-type retinas are already presented in Fig. 1C. Bars represent mean ± SEM (ANOVA, ** = *p* ≤ 0.01)

As DA supplementation lengthened the period of wild-type PER2::Luc rhythms at P8 and P30, we investigated the effect of DA supplementation in *Opn4* ^*−/−*^::*Per2* ^*Luc*^ retinal explants at both stages (Fig. 5). Interestingly, in contrast to what was observed in wild-type explants, DA supplementation (50 µM) did not lengthen the period at P8 by comparison to untreated retinas. However, a period lengthening (25.92 ± 0.24 h *p* = 0.033; Fig. 5) was observed with increased DA concentration (100 µM). In wild-type retinas, DA induced opposing changes in the phase, with a delay at P8 and an advance at P30. Moreover, DA at both concentrations did not alter the phase in *Opn4* ^*−/−*^::*Per2* ^*Luc*^ explants only at P8. These differences in the effect of DA between genotypes were no longer observed at P30. We then assessed whether differential expression of D1- (D1 and D5 receptors) or D2-like (D2 and D4) receptors could be involved in P8 *Opn4* ^*−/−*^ mouse and did not observe any difference in the relative expression of both dopaminergic receptors between the genotypes (Additional file 4: Fig. S4; *p* ≥ 0.05).

### Melanopsin has light-dependent and light-independent roles during early postnatal development

**Fig. 6:**
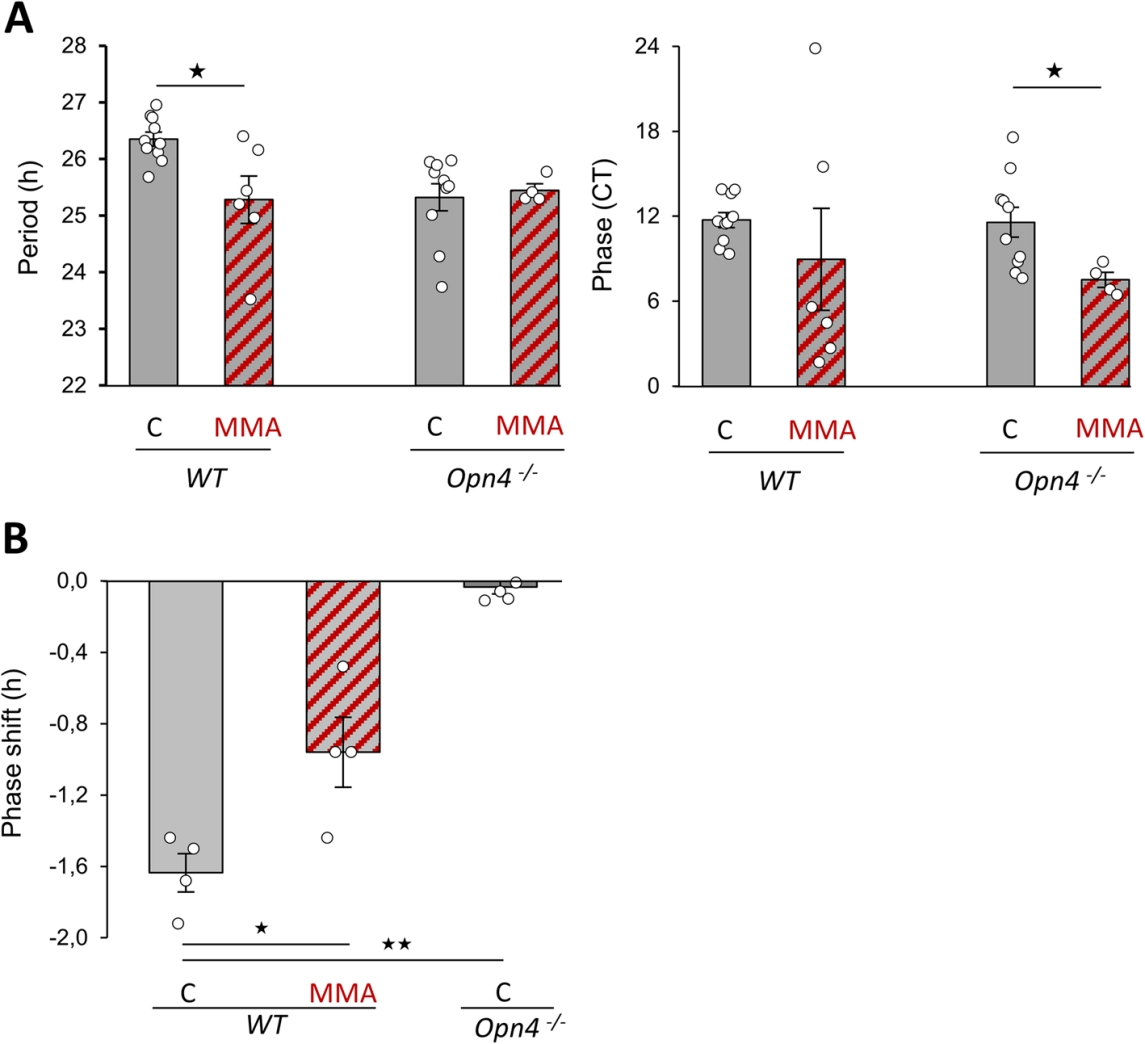
The blockade of acetylcholinergic waves shortens the period of the retinal clock. **A** Means of the period and the phase in controls (C) and retinas supplemented with mecamylamine acid (MMA) in wild-type and *Opn4* ^*−/−*^::*Per2* ^*luc*^ retinas at P8. The total numbers of retinas used were: WT: C, *n* = 10: MMA, *n* = 6; *Opn4* ^*−/−*^: C, *n* = 11; MMA, *n* = 4. Data from WT and *Opn4* ^*−/−*^ *::Per2* ^*Luc*^ control retinas are already presented in Fig. 1C. Bars represent mean ± SEM (ANOVA, * = *p* ≤ 0.05; ** = *p* ≤ 0.01; *** = *p* ≤ 0.001). **B** Mean light-induced phase shift after a 465 nm light stimulation at CT16 (30 min, 10^14^ photons/cm^2^/s) in wild-type and *Opn4*^*−/−*^::*Per2* ^*luc*^ retinas with or without MMA supplementation (MMA). Bars represent mean ± SEM (C: *n* = 4 for both genotypes; MMA, *n* = 4). Statistical differences with the C are indicated by **: *p* ≤ 0.01; ***: *p* ≤ 0.001

DA release and the intraretinal gap junction network of RGCs, including ipRGCs, have been reported to be regulated by cholinergic retinal waves during early postnatal retina development [7, 41, 42]. To assess their role in the clock, we blocked these waves in P8 *Per2* ^*Luc*^ wild-type and *Opn4* ^*−/−*^::*Per2* ^*Luc*^ explants using mecamylamine (MMA), an antagonist of cholinergic receptors (Fig. 6A). This blockade significantly shortened the period of PER2::Luc oscillations in wild-type retinal explants (control: 26.35 ± 0.12 h; treated: 25.28 ± 0.42 h; *p* = 0.026) but not in *Opn4* ^*−/−*^::*Per2* ^*Luc*^ explants (control: 25.32 ± 0.24 h; treated: 25.45 ± 0.11 h; *p* ≥ 0.05). In addition, MMA supplementation advanced the phase in *Opn4*^*−/−*^::*Per2* ^*Luc*^ retinas (control: CT 11.57 ± 1.06 h; treated: 7.50 ± 0.53 h; *p* = 0.019). However, cholinergic waves observed between P1 to P10-11 [39, 40] are followed by glutamatergic waves that rely on glutamate released from bipolar cells and activation of ionotropic glutamate receptors. Since cultured postnatal retinal explants have been reported to follow the same developmental course than in vivo, maintaining programs of cell differentiation, gene profiles and synaptogenesis [44, 45], we therefore cannot exclude a potential role of glutamatergic waves in P8 explants maintained in vitro for few days. We thus assayed their contribution by using a selective ionotropic glutamate receptors antagonist, DNQX, which blocks bipolar cell input onto retinal ganglion cells [42, 46] and retrograde input of ipRGCs to dopaminergic cells [35] (Additional file 5: Fig. S5). We found that DNQX supplementation did not alter the period (26.15 ± 0.08 h) or the phase (CT 9.52 ± 0.52) of PER2::Luc oscillations compared to untreated retinal explants (period, 25.65 ± 0.27 h; phase CT 9.82 ± 1.11; *p* ≥ 0.05). Interestingly, light detection through ipRGCs has been reported to alter the dynamic of cholinergic retinal waves in conventional RGCs [41, 42]. At P8, melanopsin- and neuropsin-expressing cells are the only functional photoreceptors [47–51]. We evaluated the light-induced phase shift of the retinal clock using 465 nm monochromatic light (10^14^ photons/cm^2^/s, 30 min, CT16) in both genotypes with and without MMA supplementation (Fig. 6B). Since the 465 nm light stimulation corresponds to around 3 log-unit decrease in neuropsin sensitivity [27], it appears unlikely that neurospin can account for the light response of the retinal clock. A significant phase delay of PER2::Luc rhythm is observed in *Per2* ^*Luc*^ wild-type explants (-1.64 ± 0.11 h, *p* = 0.04). However, this phase delay is significantly reduced after MMA supplementation (-0.96 ± 0.20 h; *p* ≤ 0.05). Furthermore, the absence of melanopsin in the *Opn4* ^*−/−*^ *::Per2* ^*Luc*^ mice prevented any light-induced phase shift of the retinal clock at this developmental age (-0.03 ± 0.04 h, *p* = 0.019).These data indicate that melanopsin is essential for the in vitro light-induced phase shift of the retinal clock during a critical developmental window and that this response is modulated by cholinergic waves.

## Discussion

In the mouse suprachiasmatic nucleus clock, fetal rhythms detectable in vivo have been reported for *Per1* mRNAs at E17 and for PER1 and PER2 proteins at E18 [52, 53] whereas in vitro studies observed PER2::LUC oscillations earlier, between E13-E15 [54, 55]. Matějů and colleagues described *Per2* expression in the inner part of the neuroblastic rat retina at P1 [56], and a recent transcriptomic study in the mouse eye showed that this gene is already expressed at E13 with a peak at P0 [26]. In our study, we found that the retina starts to oscillate with a weak amplitude at P5, marking a critical stage in the onset of the mouse retinal clock. However, we cannot rule out that distinct cell populations express PER2::Luc rhythms with different phases before P5, leading to low amplitude oscillations that are challenging to detect at the tissue level [21]. From which cells do circadian oscillations emerge? In mice, retinal neurogenesis occurs between E11 and P10, before eye-opening around P14. Early progenitor cells generate ganglion and horizontal cells, along with cone photoreceptors and some amacrines, while later progenitor cells give rise to late-born amacrine, rod, bipolar, and Müller glial cells [57–59]. Ganglion cells and cones are already present at P5, and the inner part of the neuroblastic retina gives rise to the INL, which becomes functional at the end of the first postnatal week [60]. Since PER2 is expressed and cycles at postnatal day 5, we selected another fundamental component of the molecular clock machinery, BMAL1, for several reasons. BMAL1 expression is detected in embryonic and postnatal stages with a peak at P3 [26] and it is required for important retinal functions such as visual processing, visual acuity and contrast sensitivity in the adult. Moreover, it plays a crucial role in progenitor differentiation, photoreceptor development and viability [9, 61]. Our observation of BMAL1-positive cells mainly in the inner retina at the early postnatal stages suggests that the rhythmic PER2::Luc signal mostly arises from this layer, which is consistent with data reported in the adult retina [62]. Another important question is whether the retina is able to generate rhythms without maternal influence. We found that in vitro P1 explants began to express spontaneous circadian oscillations around 4–5 days after the beginning of the culture, suggesting an autonomous developmental program that occurs within isolated retinas, independent of external signals influences. Accordingly, the endogenous period and the phase of 5-DIV P1 and P5 explants were similar, indicating that the retinal clock’s development is roughly identical between in vivo and in vitro conditions*,* at least during the initial 5 days of culture.

The current study demonstrates that the maturation of retinal clock occurs in successive steps, characterized by a progressive shortening of the endogenous period, a phase delay, and an increase in the amplitude of PER2::Luc oscillations. The period length has been closely associated with the coupling among cellular oscillators and the precision of circadian rhythms, which have been reported to decrease with period lengthening [63–65]. This gradual shortening of the period might correlate to an increased number of coupled cellular oscillators [21, 23], reflecting the maturation of cell to cell communication and their molecular components, necessary for driving coherent rhythms at the tissue level [66]. On the other hand, the phase can be reset by various intrinsic and extrinsic factors, including experimental conditions such as the time of dissection or medium changes [43]. In early postnatal days, the phase of the retinal clock may be entrained in vivo by maternal rhythms, as we have previously reported for peripheral clocks during development [67]. However, at a later stage, with eye-opening around P14, light will become a major synchronizing cue, leading to increased cellular coupling. Consistent with this, we observed an significant phase delay between P11 and P15 in wild-type explants, slightly delayed after P15 in *Opn4* ^*−/−*^ *::Per2* ^*Luc*^ retinas. How are the retinal clock features correlated during development ? The correlation between both parameters has not been thoroughly analysed in previous bioluminescence studies, either in developing or adult retinas. Under constant conditions, the phase to which a circadian clock entrains has been previously related to its endogenous period, with a shorter period usually leading to an advanced phase and a longer period is linked to a later phase. In our current study, however, we observed a negative correlation between the phase and the period through development in both genotypes. This unexpected result could be attributed to the method of phase calculation (first peak of PER2::Luc oscillations) compared to the period determination based on three complete oscillations. Under this condition, the phase measurement might reflect the time necessary for the stabilization of the retina tissue after the beginning of the culture [68].

A bundle of evidence suggests that DA might be a good candidate for mediating the coupling of cellular oscillators during development. Notably, dopaminergic cells begin to develop shortly after birth, with a rise in DA levels in the rodent retina around P2-P8 [49, 69, 70]. Moreover, DA has been shown to activate the transcriptional activity of the retinal clock [4], and processes of dopaminergic amacrine cells establish connections within the inner and outer plexiform layers and receive retrograde glutamatergic inputs from ipRGCs [33, 35, 71]. Using TH immunocytochemistry and *Th* mRNA quantification, we first confirmed that our in vitro preparation retains intact dopaminergic neurons, as previously reported in adult explants, even after 9 days of culture [62]. In wild-type C57Bl/6 mice, retinal DA does not exhibit circadian patterns, and its release is driven by spontaneous cholinergic waves during early postnatal development and by light in adulthood [7, 72]. Interestingly, DA induced opposite changes in the phase of the retinal clock, causing a delay at P8 and an advance at P30 in wild-type retinas. This may be attributed to the maturation, from P8 to P30, of retinal cells targeted by DA, which express different DA receptors, and/or to the light-induced resetting role of DA on the clock after eye-opening [1, 21, 73]. Although coupling within the adult mouse retinal clock has been reported to not rely on DA [1, 23], supplementating the medium with this neurotransmitter, acting as a chemical signal for light, induced a phase delay and period lengthening of the clock at P8. This effect was blocked by either D1R- or D2R-like antagonists. Strikingly, at this developmental stage, DA did not alter the period of *Opn4* ^*−/−*^ *::Per2* ^*Luc*^ explants, except at a higher concentration, which partially restored the shorter period. This finding suggests that the sensitivity of the retinal clock to DA is decreased in this genotype. Given that the inner retina serves as the primary source of PER2::Luc oscillations at P8 and considering the well-established regulation of gap junction conductance by DA in both adult and developmental contexts [7, 74, 75], as well as the observed effect of gap junction inhibitor on the period length (current study and [23]), we propose that during early postnatal stages, DA lengthens the clock period by reducing gap junction coupling among RGCs, ipRGCs and other inner neurons [7, 76], a modulation that may involve melanopsin. Furthermore, ipRGCs have been reported to modulate the plasticity of cholinergic wave circuitry via DA release in the neonatal retina [7, 41, 77]. These waves, in turn, regulate the network of developing ipRGCs, which are extensively coupled via gap junctions with other neurons, including ipRGCs [7, 47].

Interestingly, the blockade of cholinergic waves, but not glutamatergic waves, resulted in a shorter period and a delayed phase of the retinal clock in wild-type explants, similar to the pattern observed in the *Opn4* ^*−/−*^ explants. This suggests that melanopsin-positive cells may directly influence cholinergic amacrine cells to regulate DA release. Upon cholinergic blockade, the spontaneous release of DA may be reduced, promoting robust electrical coupling between inner neighboring cells. This synchronization of the cellular network ultimately results in a shortening of the period in wild-type mice. Notably, the disruption of cholinergic waves did not affect these clock parameters in the *Opn4* ^*−/−*^ retinas, indicating that melanopsin may modulate period length through cholinergic waves under dark conditions. However, the underlying reason for the persistently shorter period in the adult *Opn4* ^*−/−*^ retinas remains to be delineated. It is worth noting that light has been reported to increase cholinergic wave duration through melanopsin [41] without affecting their frequency [78]. Our findings indicate that melanopsin is necessary for the light-induced phase shift of the retinal clock at a developmental stage when rods are not yet functional. This finding differs from our previous work in adults, where we demonstrated that rods, but not melanopsin, are required for the light response of the clock [27]. This highlights the differential contribution of melanopsin through the time course of development. Furthermore, the light-induced phase shift is significantly reduced, although not abolished, after blocking cholinergic waves. ipRGCs have been reported to signal retrogradely to various types of amacrine cells, releasing DA, serotonin, neuropeptide Y or nitric oxide synthase [32, 34–36, 79, 80]. Given that all retinal neurons express the clock machinery and that the PER2::Luc signal predominantly originates from the inner retina at P8, we can presume that the inner cellular oscillators are primarily responsible for the light-induced phase shift, with cholinergic amacrine cells accounting for around 42% of the overall phase shift. Several studies have demonstrated that ipRGCs subserve different roles in neonatal mice, compared to adults [50, 81, 82]. Our findings shed light on a previously unrecognized function of melanopsin in modulating the retinal clock through cholinergic waves under both dark and light conditions during early development. The precise mechanism underlying this modulation is still not fully understood, but may be related to the significant decrease in melanopsin expression and ipRGCs density after P8 in the wild-type mice [47, 48, 51].

## Conclusions

Our study comprehensively characterizes the development and maturation of the mouse retinal clock, shedding light on the previously unknown functions of DA and melanopsin in the clock’s endogenous functioning and light response through the regulation of cholinergic waves during development. This study highlights the importance of understanding the diverse and potent mechanisms that impact the development of the retinal clock network.

## Methods

### Animals

Male and female C57BL/6 J *Per2* ^*Luc*^ [83] and *Opn4* ^*−/−*^*::Per2* ^*Luc*^ mice were housed in a temperature-controlled room (23 ± 1 °C), under 12 h light/12 h dark cycle (12L/12D, light intensity around 200–300 lx) with food and water ad libitum. All animal procedures were in strict accordance with current national and international regulations on animal care, housing, breeding, and experimentation and approved by the French Ministry of Higher Education, Research and Innovation (APAFIS #19,976–2,019,032,610,179,225). All efforts were made to minimize suffering. Animals were used at E18, P1, P5, P8, P11, P15, P30, and in 2 month-old adults. The day of birth corresponded to P1.

### Retinal explant culture

For E18, *Per2* ^*Luc*^ pregnant mice were killed by cervical dislocation, and embryos were isolated. Animals were sacrificed by decapitation between P1 and P8 and cervical dislocation from P11. All animals were sacrificed one hour before light offset at zeitgeber time 11 (ZT11). Eyes were enucleated and placed on ice in Hank’s balanced salt medium (HBSS; Invitrogen). Retinas were gently isolated from the rest of the eyecup and flattened, ganglion cell layer up, on a semi-permeable (Millicell) membrane in a 35 mm culture dishes (Nunclon) containing 1.2 mL Neurobasal-A (Life Technologies) with 2% B27 (Gibco), 2 mM L-Glutamine (Life Technologies) and 25 U/mL antibiotics (Penicillin/Streptomycin, Sigma), incubated at 37 °C in 5% CO2 for 24 h. From this step onwards, all manipulations of explants were performed under dim red light. After 24 h, at the projected ZT12, retinas were transferred to 1.2 ml of 199 medium (Sigma), supplemented by 4 mM sodium bicarbonate (Sigma), 20 mM D-glucose (Sigma), 2% B27, 0.7 mM L-Glutamine, 25 U/mL antibiotics (Penicillin/Streptomycin, Sigma) and 0.1 mM Luciferin (Perkin). Culture dishes were sealed and placed in a Lumicycle (Actimetrics, Wilmette, IL, USA) to record the global emitted bioluminescence. All medium changes were performed under dim red light.

### Pharmacological treatments

When applicable, 199 medium was supplemented with different molecules that remained in the medium throughout the entire recording without further medium changes: DA (50 and 100 µM), alpha methyl-L-tyrosine, an inhibitor of DA synthesis (L-AMPT, 100 µM), reserpine, an inhibitor of vesicular DA uptake (Res, 10 µM), apomorphine, a non-selective DA agonist (Apo, 50 µM), a DA D1R antagonist SCH39166 (50 µM), a DA D2R antagonist L741626 (25 µM), carbenoxolone, a non-selective gap junction blocker (CBX, 100 µM), mecamylamine, an acetylcholine receptor antagonist (MMA, 100 µM) and 6,7-Dinitroquinoxaline-2,3-dione disodium salt (DNQX, 20 µM), a selective ionotropic glutamate receptor antagonist.

### Light-induced phase shift

P8 retinal explants from *Per2* ^*Luc*^ and *Opn4* ^*−/−*^ *::Per2* ^*Luc*^ mice were exposed to a 465 nm monochromatic light pulse (30 min, 10^14^ photons/cm^2^/s, CT16) using bright LED light sources (Superbrightleds) with or without MMA supplementation. To avoid putative bias linked to retina development in culture, light stimulation was applied on the first PER2::Luc oscillation at CT16. Briefly, CT16 was determined based on the time of occurrence of the trough of the first PER2::Luc oscillation using the beginning of culture (CT12) as a reference as previously described [27, 43]. Retinas from the same animal were either light-stimulated or used as a dark control. Phase shifts following light stimulation or MMA application were calculated as the difference between the trough time of the cycle after treatment compared to the trough time of the control retina from the same animal, as determined by Lumicycle Analysis software.

### Quantitative RT-PCR

To quantify DA receptors, 2 retinas of wild-type and *Opn4* ^*−/−*^ mice at P8 and P30 were dissected at ZT11, pooled, and stored at -80 °C until RNA extraction and quantification. For *Th* quantification, retinas were collected at CT0 and CT12 or after 2 days of culture at the trough or the peak of PER2::Luc oscillations. Total RNAs were extracted using Trizol reagent (Invitrogen) and reverse transcribed using random primers and MMLV Reverse Transcriptase (Invitrogen). Real-time RT-PCR was then performed on a LightCycler™ system (Roche Diagnostics) using the light Cycler-DNA Master SYBR Green I mix. Hypoxanthine ribosyl-transferase (*Hprt*) was used for the internal standardization of target gene expression. The efficiency and the specificity of the amplification were controlled by generating standard curves and carrying out melting curves. Relative transcript levels of each gene were calculated using the second derivative maximum values from the linear regression of cycle number versus log concentration of the amplified gene. Primer sequences were: *Hprt* sens ATCAGTCAACGGGGGACATA and reverse AGAGGTCCTTTTCACCAGCA; *D1R* sens CAGCCTTCATCCTGATTAGCGTAGGCG and reverse CTTATGAGGGAGGATGAAATGGCG*; D2R* sens CAGTGAACAGGCGGAGAATG and reverse CAGGACTGTCAGGGTTGCTA; *D4R* sens CGTCTCTGTGACACGCTCATTA and reverse CACTGACCCTGCTGGTTGTA; *D5R* sens CATCCATCAAGAAGGAGACCAAGG and reverse CAGAAGGGAACCATACAGTTCAGG, *Th* sens GAAGGGCCTCTATGCTACCC and reverse GGCATGACGGATGTACTGTG.

### Immunohistochemistry

Retinal explants from *Per2* ^*Luc*^ mice were cultured as previously described and fixed for 24 h in 4% paraformaldehyde (PFA) after 2 days in vitro (2-DIV). Retinas were blocked with 1% bovine serum albumin (BSA) and 0.3% Triton X-100 in 0.1 M phosphate buffered saline (PBS) for 2 h and then incubated in the sheep anti-TH (1/200, Millipore) antibody at 4 °C for 24 h. After rinsing, a secondary incubation was performed for 2 h with the secondary anti-sheep biotinylated IgG (1/200, Vector Laboratories, Burlingame, USA), rinsed twice and incubated with Cy3-conjugated streptavidin (1/500, Jackson Immunoresearch laboratories). Samples were then mounted and coverslipped using Vectashield (Vector Laboratories). For BMAL1 staining, pups at P3, P5 and P8 (*n* = 3 for each genotype) were rapidly anesthetized and perfused intracardially with warm heparinized saline followed by PFA fixative at 4 °C. The eyes were removed and post-fixed overnight in the same fixative at 4 °C, then rinsed in 0.1 M phosphate buffer (PB, pH 7.4). Eyes were cryoprotected in 30% sucrose in PB for 24 h and sections of the retina were made at 20 µm on a cryostat. Retinal sections were incubated overnight with the rabbit antibody BMAL1 (1/500, Novus Biological), followed by a biotinylated goat anti-rabbit for 2 h (1/200; Vector Laboratories) and a Cy3-conjugated streptavidin (1/500, Jackson Immunoresearch laboratories). Retinal sections were then counsterstained with DAPI (1/10000, Invitrogen) for 5 min. Micrographs were obtained using Leica confocal microscopy.

### Data analysis

Rhythm onset was defined when the first complete bioluminescence oscillation was observed (first trough and peak clearly distinguishables). The period and the amplitude of PER2::Luc oscillations were determined using SigmaPlot 12.5 software by fitting a linearly detrended sinusoidal curve oscillating around a polynomial baseline to the first three complete oscillations from each sample as previously described [23, 27, 43]. The equation used was:

$$y\left(x\right)=\left(a-b*x\right)*\mathrm{sin}\left(2*\pi *\frac{x+\varphi }{\tau }\right)+(c+d*x+e*{x}^{2}+g*{x}^{3})$$

where a is the amplitude (difference between the peak and the mesor values in the detrented time series data), τ is the period (duration of a complete circadian cycle, time peak to peak), and φ the phase. The mesor represents the circadian rhythm-adjusted mean based on the parameters of the sinusoidal function fitted to the raw data. The phase referred to the circadian time of the fitted peak of the first oscillation in the current manuscript, and was calculated based on a time reference (CT12), which corresponds to the time of medium change corrected by the endogenous period [27, 43].

### Statistics

Statistical analyses (Statistica Software) were performed using non parametric Kruskal Wallis one-way ANOVA followed when significant by the Mann–Whitney post-hoc test in order to compare the period, the phase and the amplitude of PER2::Luc signals, pharmacological treatments, and the relative expression of DRs and *Th* mRNA during development in *Per2* ^*Luc*^ mice. Non parametric Kruskal Wallis two-ways ANOVA, followed by the Mann–Whitney post-hoc test, were used to compare the clock parameters between *Per2* ^*Luc*^ and *Opn4* ^*−/−*^ *::Per2* ^*Luc*^ mice during development. Data are represented as mean ± SEM, with consideration of *p* ≤ 0.05 as the threshold of statistical significance.

## Supplementary Information

**Additional file 1: Fig. S1.** Representative bioluminescence recording of PER2::Luc retinal explants at embryonic day 18. No oscillations of PER2::Luc were detected in cultured retinal explants even after 6 days in culture.

**Additional file 2: Fig. S2.** Scatter plots showing the relationship between retinal clock parameters in Per2^luc^and Opn4^-/-^::Per2^luc^retinas during development. A. The phase versus the endogenous period of the retinal circadian clock shows a negative, linear correlation in both genotypes with no significant change in the slope between genotypes. B. The period versus the amplitude did not show a significant correlation between the period and the amplitude for both genotypes and across the different developmental stages.

**Additional file 3: Fig. S3. **Dopamine did not influence the onset of the spontaneous oscillations of PER2::Luc in vitro. A. Representative bioluminescence recording of PER2::Luc 5 days in vitro P1 cultured explants supplemented with DA. The dotted black rectangles correspond to the analysis windows including the 3 first complete oscillations. B. Comparison of the means of the endogenous period, the phase, and the amplitude of 5-DIV P1, 5-DIV + DA and P5 retinal explants. The total numbers of retinas used were: 5-DIV P1, *n*=5; 5-DIV + DA, *n*=4; P5, *n*=6. Bars represent mean ± SEM.

**Additional file 4: Fig. S4. **The relative expression of D1- and D2-like dopaminergic receptors are similar between wild-type and Opn4^-/^^-^retinas at P8. Means of the relative expression of D1-like and D2-like receptors in the retinas of wild-type and Opn4^-/-^mice. The total numbers of retinas used were: WT, *n*=6 ; Opn4^-/-^, *n*=7. Bars represent mean ± SEM.

**Additional file 5: Fig. S5. **The blockade of glutamatergic waves has no effect on the period and the phase of the retinal clock. A. Means of the period and the phase in controland retinas supplemented with 6,7-Dinitroquinoxaline-2,3-dione disodium saltin P8 explants from wild-type Per2^luc^ mice. The total numbers of retinas used were: C, *n*=4; DNQX, *n*=5. Bars represent mean ± SEM.

**Additional file 6: Table S1. **Raw data.

## Data Availability

All data generated or analyzed during this study are included in this published article and its supplementary information files (Additional file 1: Fig. S1, Additional file 2: Fig. S2, Additional file 3: Fig. S3, Additional file 4: Fig. S4, Additional File 5: Fig. S5). Raw data for this study are provided in Additional file 6: Table S1.

## Abbreviations

Apo: Apomorphine
BSA: Bovine serum albumin
CBX: Carbenoxolone
CT: Circadian time
DA: Dopamine
D1R: D1 receptor
D2R: D2 receptor
D4R: D4 receptor
D5R: D5 receptor
DR: Dopaminergic receptor
DNQX: 6,7-Dinitroquinoxaline-2,3-dione disodium salt
E: Embryonic
GCL: Ganglion cell layer
*Hprt*: Hypoxanthine ribosyl-transferase
INL: Inner nuclear layer
ipRGCs: Melanopsin intrinsically photosensitive ganglion cells
L-AMPT: Alpha methyl-L-tyrosine
MMA: Mecamylamine
*Opn4*^*−*^^*/*^^*−*^: Melanopsin knockout mouse
P: Postnatal
PER1: Period 1 protein
PER2: Period 2 protein
PER2::Luc: PERIOD2 luciferase
PB: Phosphate buffer
PBS: Phosphate buffered saline
PFA: Paraformaldehyde
Res: Reserpine
RGCs: Retinal ganglion cells
TH: Tyrosine hydroxylase protein
*Th*: Tyrosine hydroxylase gene
ZT: Zeitgeber time
5-DIV: 5 Days in vitro
