# Supplementary figures and images for: Dopamine modulates the retinal clock through melanopsin-dependent regulation of cholinergic waves during development
Source: BMC Biol. 2023 Jun 26;21:146. doi: 10.1186/s12915-023-01647-6 (PMC10294308; doi:10.1186/s12915-023-01647-6)

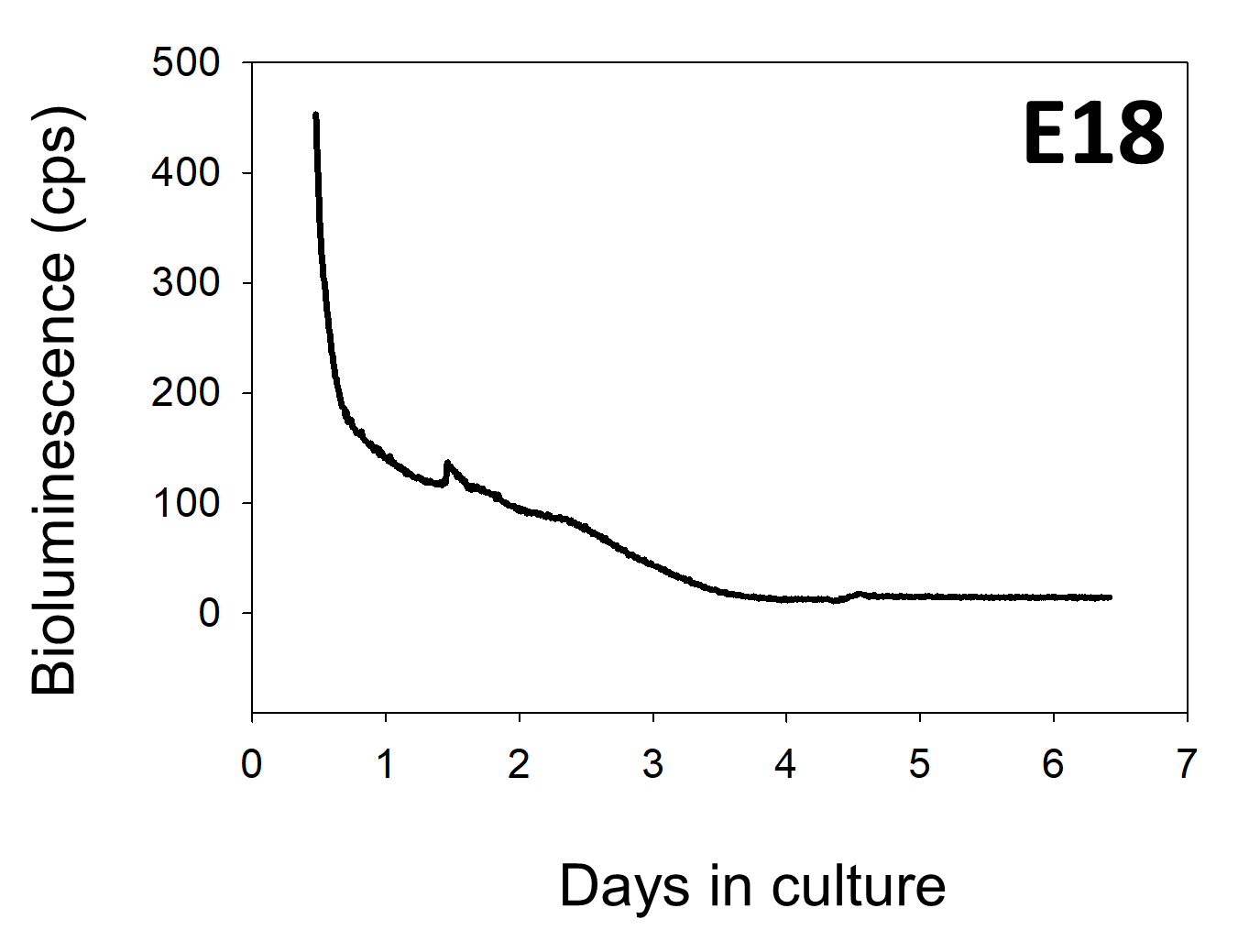

Supplement: Supplementary file 1 — Additional file 1: Fig. S1. Representative bioluminescence recording of PER2::Luc retinal explants at embryonic day 18. No oscillations of PER2::Luc were detected in cultured retinal explants even after 6 days in culture. [file 12915_2023_1647_MOESM1_ESM.tif]

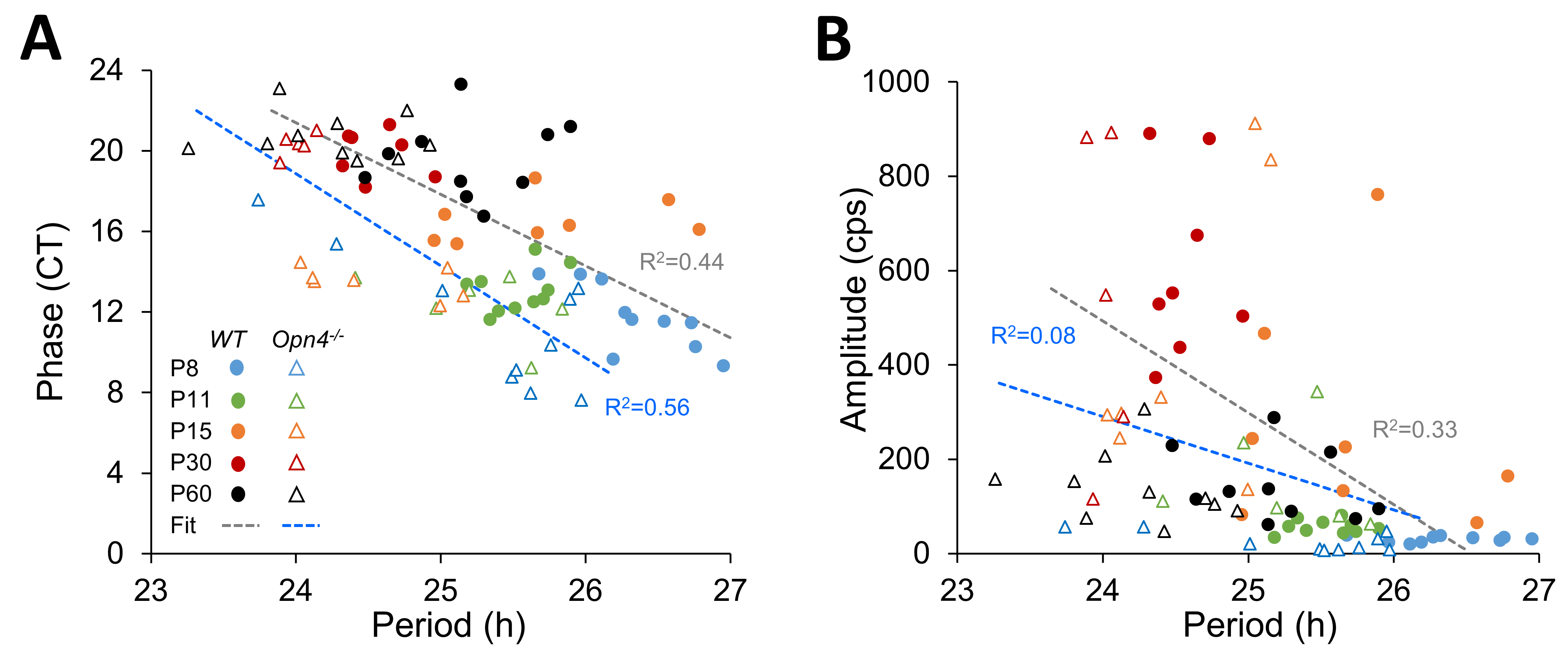

Supplement: Supplementary file 2 — Additional file 2: Fig. S2. Scatter plots showing the relationship between retinal clock parameters in Per2lucand Opn4-/-::Per2lucretinas during development. A. The phase versus the endogenous period of the retinal circadian clock shows a negative, linear correlation in both genotypes with no significant change in the slope between genotypes. B. The period versus the amplitude did not show a significant correlation between the period and the amplitude for both genotypes and across the different developmental stages. [file 12915_2023_1647_MOESM2_ESM.tif]

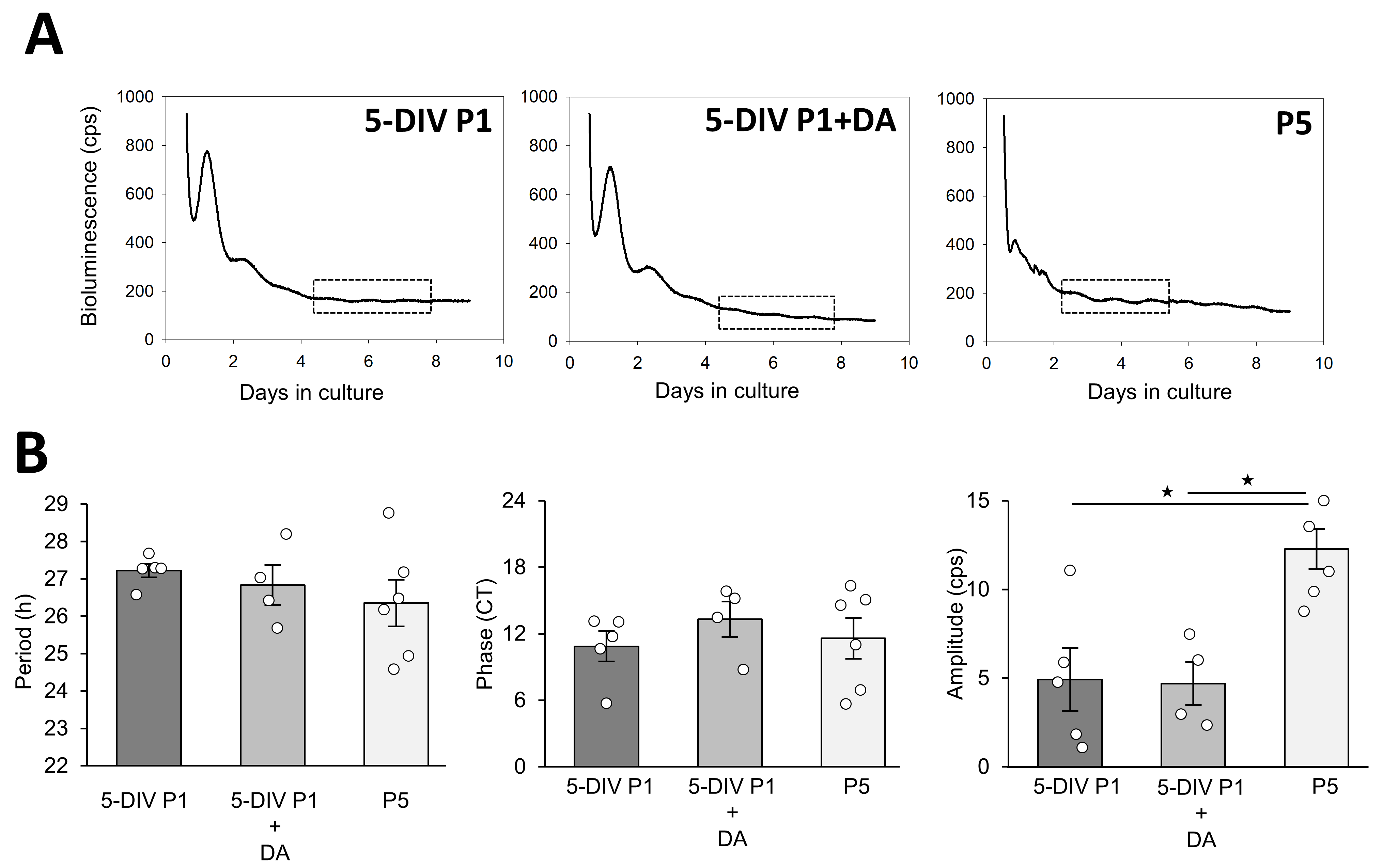

Supplement: Supplementary file 3 — Additional file 3: Fig. S3. Dopamine did not influence the onset of the spontaneous oscillations of PER2::Luc in vitro. A. Representative bioluminescence recording of PER2::Luc 5 days in vitro P1 cultured explants supplemented with DA. The dotted black rectangles correspond to the analysis windows including the 3 first complete oscillations. B. Comparison of the means of the endogenous period, the phase, and the amplitude of 5-DIV P1, 5-DIV + DA and P5 retinal explants. The total numbers of retinas used were: 5-DIV P1, n=5; 5-DIV + DA, n=4; P5, n=6. Bars represent mean ± SEM. [file 12915_2023_1647_MOESM3_ESM.tif]

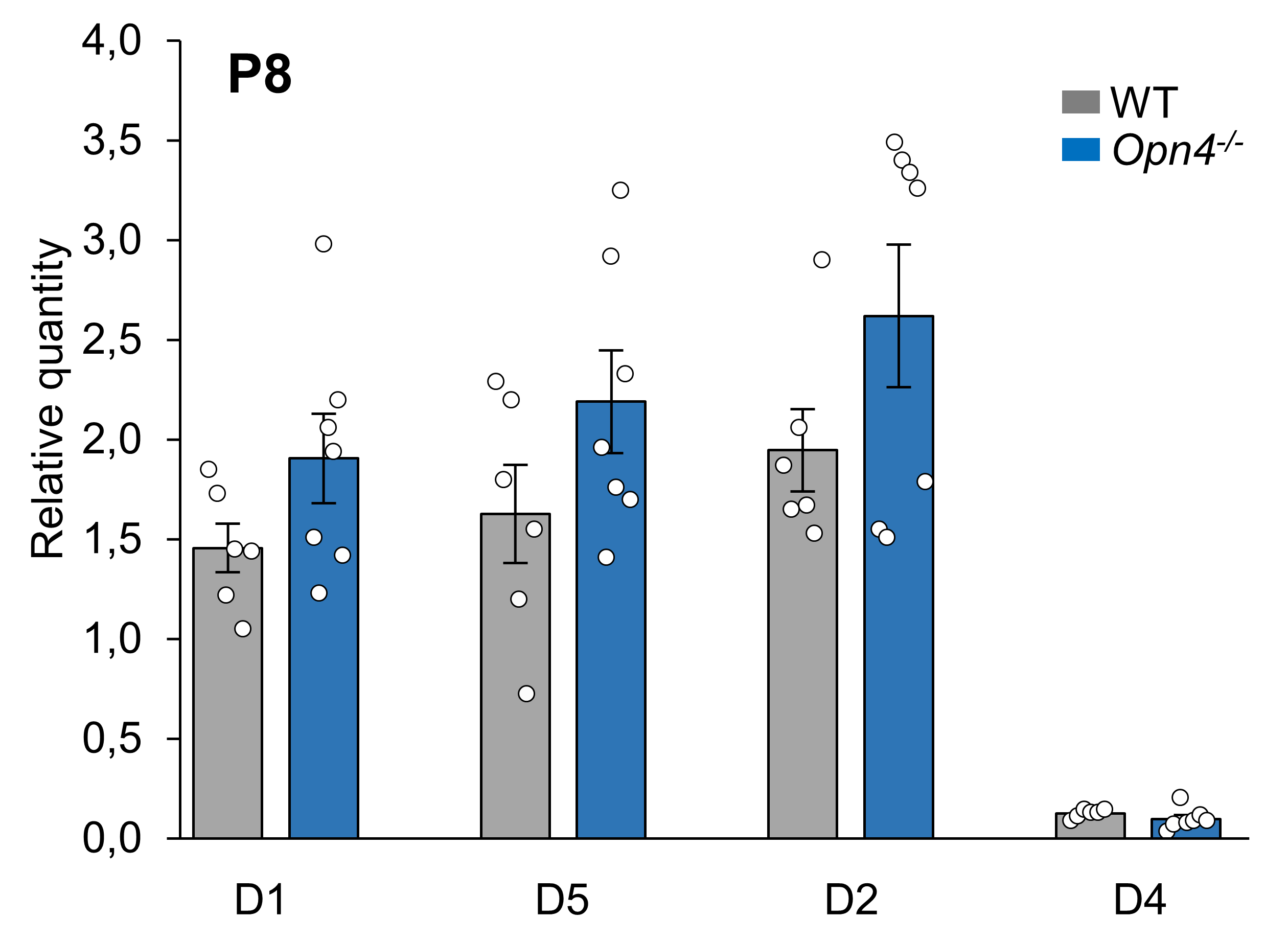

Supplement: Supplementary file 4 — Additional file 4: Fig. S4. The relative expression of D1- and D2-like dopaminergic receptors are similar between wild-type and Opn4-/-retinas at P8. Means of the relative expression of D1-like and D2-like receptors in the retinas of wild-type and Opn4-/-mice. The total numbers of retinas used were: WT, n=6 ; Opn4-/-, n=7. Bars represent mean ± SEM. [file 12915_2023_1647_MOESM4_ESM.tif]

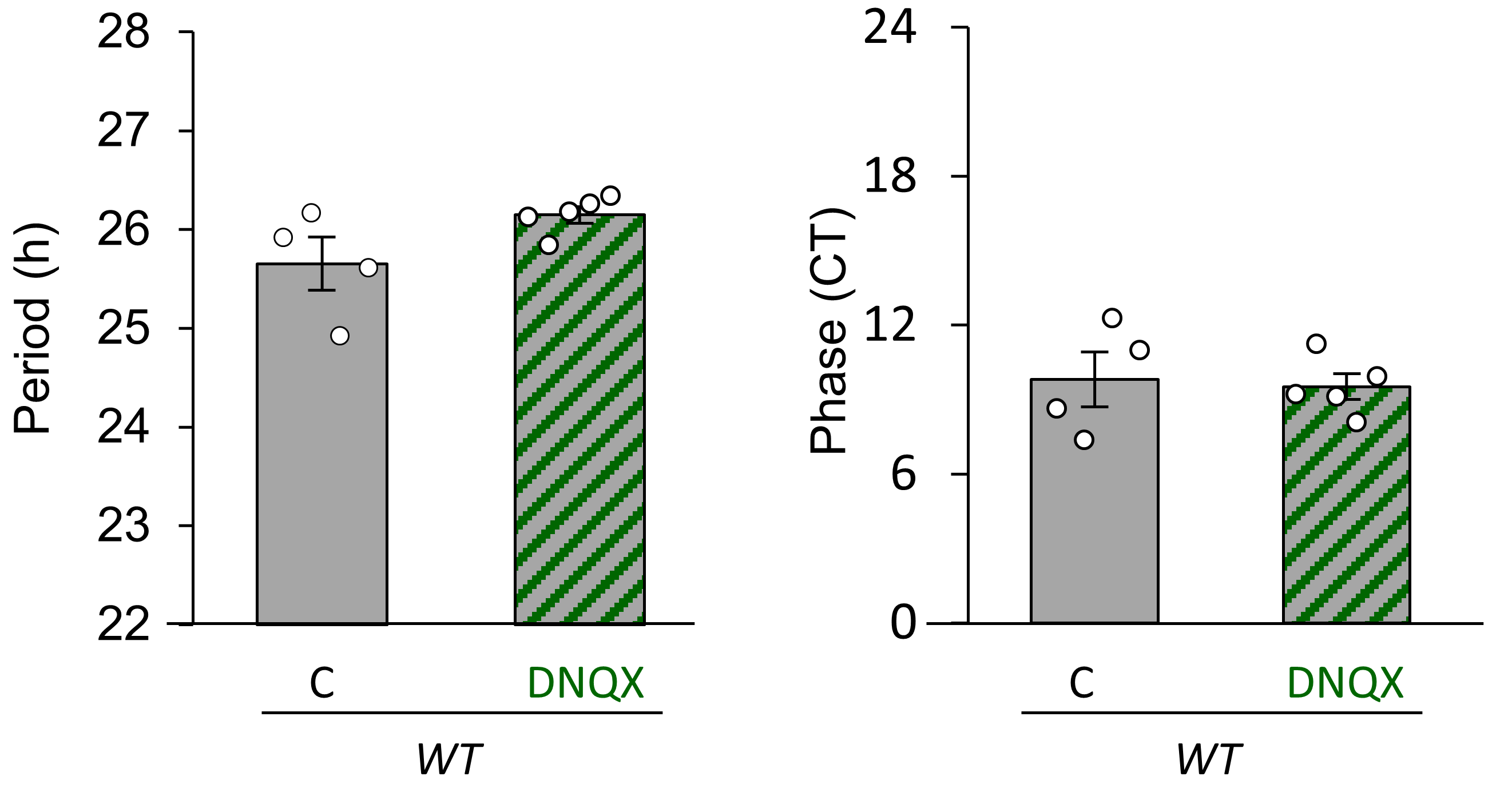

Supplement: Supplementary file 5 — Additional file 5: Fig. S5. The blockade of glutamatergic waves has no effect on the period and the phase of the retinal clock. A. Means of the period and the phase in controland retinas supplemented with 6,7-Dinitroquinoxaline-2,3-dione disodium saltin P8 explants from wild-type Per2luc mice. The total numbers of retinas used were: C, n=4; DNQX, n=5. Bars represent mean ± SEM. [file 12915_2023_1647_MOESM5_ESM.tif]
